# DNA Methylation - and Telomere - Based Biological Age Estimation as Markers of Biological Aging in Donors Kidneys

**DOI:** 10.3389/fmed.2022.832411

**Published:** 2022-03-23

**Authors:** Sofia Pavanello, Manuela Campisi, Paolo Rigotti, Marianna Di Bello, Erica Nuzzolese, Flavia Neri, Lucrezia Furian

**Affiliations:** ^1^Occupational Medicine, Department of Cardiac, Thoracic, and Vascular Sciences and Public Health, University Hospital of Padova, Padova, Italy; ^2^Kidney and Pancreas Transplantation Unit, Department of Surgery, Oncology and Gastroenterology, University Hospital of Padova, Padova, Italy

**Keywords:** kidney transplantation, DNA methylation age, telomere length, age acceleration, kidney rejuvenation, biological age

## Abstract

The biological age of an organ may represent a valuable tool for assessing its quality, especially in the elder. We examined the biological age of the kidneys [right (RK) and left kidney (LK)] and blood leukocytes in the same subject and compared these to assess whether blood mirrors kidney biological aging. Biological age was studied in *n* = 36 donors (median age: 72 years, range: 19–92; male: 42%) by exploring mitotic and non-mitotic pathways, using telomere length (TL) and age-methylation changes (DNAmAge) and its acceleration (AgeAcc). RK and LK DNAmAge are older than blood DNAmAge (RK vs. Blood, *p* = 0.0271 and LK vs. Blood, *p* = 0.0245) and RK and LK AgeAcc present higher score (this mean the AgeAcc is faster) than that of blood leukocytes (*p* = 0.0271 and *p* = 0.0245) in the same donor. TL of RK and LK are instead longer than that of blood (*p* = 0.0011 and *p* = 0.0098) and the increase in Remuzzi-Karpinski score is strongly correlated with kidney TL attrition (*p* = 0.0046). Finally, blood and kidney TL (*p* < 0.01) and DNAmAge (*p* < 0.001) were correlated. These markers can be evaluated in further studies as indicators of biological age of donor organ quality and increase the usage of organs from donors of advanced age therefore offering a potential translational research inkidney transplantation.

## Introduction

Organ failure represents a dramatic socio-economical burden worldwide which prevalence is likely to increases sharply with population aging. The ideal therapeutic solution is represented by the transplantation of an allogeneic equivalent obtained by a human donor. However, there is a dramatic mismatch between the number of patients in transplantation list and the effective availability of donor's organs (1). Strategies to face this issue have led the transplant scientific community to progressively expand the eligibility criteria for donors to include the elderly (2).

Within the context of kidney transplantation in which organ shortage remains a problem, donor's age is however one of the main factors influencing the decision of accepting the kidneys (1, 2). Kidneys from aged people may have chronic damages, which make them less efficient in the function recovery upon the ischemia and reperfusion injury (3, 4). Furthermore, the assessment of the kidney relies also on the histology (i.e., Remuzzi score), which reflects a morphological feature (5).

Although the chronologicalage of the donor can influence the quality of the kidney to be transplanted, it may not be a reliable indicator of the rate of physiological breakdown of the body or of the organs, as people do not age at the same rate. Genetic and environmental factors can impact on biological aging, causing different aging trajectories and health outcomes in each person (6–8). In addition, a different rate of aging seems to occur also within the same subject in each system and organ (9). In our previous studies, we demonstrated that the biological age of cardiac tissues procured from deceased donors were consistently younger than their chronological age (10). Therefore, it can be postulated that even the kidney may have a different aging profile. Defining the biological age of kidney may contribute to supporting this process.

The biological age is not that indicated on the identity card but is written on the DNA. Growing indications have shown that telomere length (TL) and age-correlated DNA methylation changes in certain CpG loci (DNAmAge) are early hallmarks of biological aging, and may be primary indicators of cellular dysfunction and in age-related disorders (9, 10). TL, the non-coding DNA sequences that cap chromosomes and shorten at each cell division, measures mitotic or replicative cellular aging (11). DNA methylation age (DNAmAge) is an emerging epigenetic marker of non-mitotic cellular aging (12, 13), assessed through the analysis of methylation at a specific subset of cytosine-guanine dyads (CpG), which showed a strong correlation with the chronological age (14–17). We recently automated the method proposed by Zbieć-Piekarska et al. (16) to increase the efficiency and rapidity, maintaining high prediction accuracy (9, 10). The development of these biomarkers has led to the definition of an “epigenetic clock” theory of aging; the difference between DNAmAge and chronological age defined as “age acceleration” (AgeAcc) (18) is indicative of altered biological functions (13) and elevated risk for morbidity and mortality (19). Findings on TL (20) and DNAmAge (14–16) are quite exclusively based on DNA from blood circulating leukocytes, as they represent an easily available DNA source.

Studies comparing biological age indicators measured in different tissues of the same subject are however in most of cases on TL measures made on cadavers, donors elderly patients, and the measurements seem correlated (21–23). There have been instead few studies investigating correlation of DNAmAge in different tissues (14, 24). Of particular note is the Horvath epigenetic clock that was developed to be applicable across human tissues (14), but correlations were not made between blood and tissues, in the same subject. From a translational perspective, it remains to be clarified whether DNAmAge in lymphocytes mirror that in the different tissues/organs in healthy donors.

Furthermore, the biological age of an organ may then represent a valuable tool for the assessment of its quality, as it could correlate more closely to its functional reserve, and predict the transplantation outcome more accurately than the present evaluation methods.

The aim of our study was:

1) To determine the biological age of the kidneys by measuring the mitotic (TL) and the non-mitotic epigenetic age (DNAmAge) of renal cells collected from kidney samples and to compare it with the Remuzzi score with the purpose of defining a biomarker for organ quality assessment.
2) To compare the DNAmAge of peripheral blood leukocytes and kidneys, in order to establish whether blood may be an accurate indicator of kidney biological age.

## Materials and Methods

### Study Design: Kidney Procurement, Sampling of Donor's Tissue and Blood, and Data Collection

Over a time span of 22 months (from March 2019 to January 2021) renal true-cut biopsies and blood samples were obtained in the same time from 36 deceased kidney donors for whom the procurement was performed by the surgical team of Kidney and Pancreas Transplantation Unit - Department of Surgical, Oncological and Gastroenterological Sciences, University Hospital of Padua. The donors included in the study were those for whom a renal biopsy was deemed clinically indicated either for the assessment of chronic damage (2) or for the presence of acute kidney injury. Donors' Characteristics (median age: 72 years, range: 19–92; male: 42%) are summarized in Table 1. Details of the donor's kidney biopsy procedure are reported in the Supplementary Materials.

**Table 1:** Donors' Characteristics.

**N**	**36**
**Age (y)**	72 (19–92)
Gender	
Male (*n*, %)	15 (42%)
Female (*n*, %)	21 (58%)
Smoking	11 (31%)
Comorbidities	
Arterial Hypertension (PAH)	21 (58%)
Diabetes	4 (11%)
Other	4 (11%)
Cause of death	
Ictus	10 (28%)
Cranial trauma	9 (25%)
Subarachnoid hemorrhage (SAH)	14 (39%)
Cardiac arrest	2 (5%)
Other	1 (3%)
Blood parameters	
Leukocytes (*n* * 10^9^/L)	12.395 (2.96–32.22)
Renal function	
Creatinine (mg/dL)	0.88 (0.41–1.85)
Histological evaluation of kidneys (Remuzzi Score)	
0 ≤ Score ≤ 3	12 (26%)
4 ≤ Score ≤ 6	26 (57%)
Score ≥ 7	8 (17%)
TL	
Right kidney	1.18 (0.93–1.63)
Left kidney	1.25 (0.76–1.99)
Blood	1.15 (0.38–2.05)
DNAmAge	
Right kidney	69 (29–80)
Left kidney	66 (23–79)
Blood	65 (13–78)

*Data are expressed in median (range) or number (percentage)*.

Blood samples (3–4 ml) from the donors were collected in K3EDTA and PAXgene tubes (BD Biosciences, Milano, Italy). The tru-cut biopsies were placed in all protected tissue reagent-RNA Later (Qiagen, Milano, Italy) for DNA/RNA stabilization. All collected samples were then, transferred to our laboratory of Genomic and Environmental Mutagenesis (Department of Cardiac, Thoracic, and Vascular Sciences and Public Health, University-Hospital of Padua) for genetic and epigenetic analyses and stored at −20°C, until analyses were performed. Our Local Ethical Committee, which is named the Ethical Committee for Clinical Trials of the Province of Padova, approved the study (protocol number 2246P) in accordance with principles of the Helsinki Declaration, allowing a waiver from consent. All methods were carried out in accordance with relevant guidelines and regulations.

We collected data on the following donor characteristics: age, gender, smoking, comorbidities, cause of death, blood parameters and renal function. We also recorded the histological Remuzzi-Karpinski score of the kidneys when available for clinical necessity as for the Nord Italian Transplant programm (NITp) algorithm for allocation to single or dual transplantation (2).

### DNA Extraction From Blood and Tissue Samples

DNA extraction was performed on all samples of whole blood and renal biopsies using an automated QIAcube System according to the DNAeasy Blood and Tissue kit procedure (Qiagen, Milano, Italy) as previously described (10). Details of DNAmAge analysis are reported in the Supplementary Materials.

### DNAmAge Analysis

DNAmAge was assessed by analyzing the methylation levels of five selected markers in genomic DNA using bisulfite conversion and Pyrosequencing methodology as previously described (9, 10, 17). Twenty percent of the samples were analyzed in two different days to verify the reproducibility of our results and the coefficient of variation (CV) in replicate pyrosequencing runs was 1.7 %. Details of DNAmAge analysis are reported in the Supplementary Materials.

### AgeAcc Evaluation

AgeAcc was assessed for both renal tissue and blood leukocytes of each donor. AgeAcc was estimated as the difference between the DNAmAge and the chronological age of the donors.

### TL Analysis

TL was measured in genomic DNA by quantitative Real-Time PCR by estimating the ratio of telomere repeat copy number (T) to single nuclear copy gene (S) in experimental DNA samples relative to the T/S ratio of a reference pooled sample as previously reported (25–27). The average of CV for the T/S ratio of samples analyzed over three consecutive days was 9%, which was similar to the original method (28). Details of TL analysis are reported in the Supplementary Materials.

### Sample Size Estimation

Estimating that a significant correlation would be in the order of r = 0.80, we calculated that the sample to obtain statistical significance (α 0.01) should be *n* = 15 (power 0.9).

### Statistical Analysis

Statistical analyses were performed with StatsDirect software. Data are expressed as median, minimum and maximum values unless otherwise specified. Values of TL, DNAmAge and AgeAcc in Kidneys (renal biopsies) and blood, of the same patient, were compared by (two-tailed) paired *T*-test, while comparison between all samples in the two groups was also made using Mann-Whitney U Test. Correlation was evaluated by simple linear regression models (Kendall's rank correlation) in order to provide a measure of the strength of dependence between two variables. Results were considered significant when a *p* value of < 0.05 was obtained.

## Results

### Biological Age of Kidneys and Blood Leukocytes Determined by DNAmAge, AgeAcc and TL

**Figure 1:**
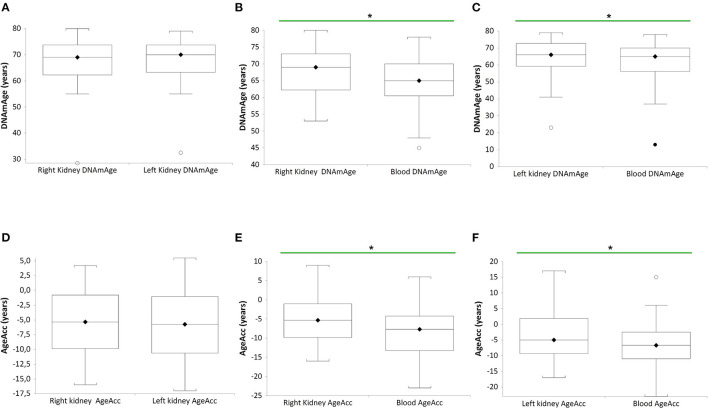
DNAmAgeand AgeAcc of both kidneys (right and left) and blood leukocytes in the same donors (*n* = 27). Box plots show levels of DNAmAge in right and left kidneys (RK and LK) **(A)**, in RK and blood **(B)**, and in LK and blood **(C)** of the same donors. In box plots, the boundary of the box closest to the x-axis indicates the 25th percentile, the line within the box marks the mean, and the boundary of the box farthest from the x-axis indicates the 75th percentile. Whiskers (error bars) above and below the box indicate the 95 and 5th percentiles. In **(B)**, the horizontal bar with asterisk indicates the significant comparison between RK DNAmAge and paired blood DNAmAge of the same donor (n = 27) (^*^Paired *t*-test: median 69 years vs. median 65 years; *p* = 0.0271). In **(C)**, the horizontal bar with asterisk indicates the significant comparison between LK DNAmAge and paired blood DNAmAge of the same donor (*n* = 27) (^*^Paired *t*-test: median 69 years vs. median 65 years; *p* = 0.0245). Box plots show levels of AgeAcc in right and left kidneys (RK and LK) **(D)**, in RK and blood **(E)**, and in LK and blood **(F)** of the same donors. In box plots, the boundary of the box closest to the x-axis indicates the 25th percentile, the line within the box marks the mean, and the boundary of the box farthest from the x-axis indicates the 75th percentile. Whiskers (error bars) above and below the box indicate the 95 and 5th percentiles. In **(E)**, the horizontal bar with asterisk indicates the significant comparison between RK AgeAcc and paired blood AgeAcc of the same donor (*n* = 27) (^*^Paired *t*-test: median−5 years vs. median−8 years; *p* = 0.0271). In **(F)**, the horizontal bar with asterisk indicates the significant comparison between LK AgeAcc and paired blood AgeAcc of the same donor (*n* = 27) (^*^Paired *t*-test: median−6 years vs. median−7 years; *p* = 0.0245).

In Figure 1, DNAmAge of the right (RK) (*n* = 27 paired RK vs blood DNAmAge donors (B), paired *t*-test: median 69 years vs. median 65 years; *p* = 0.0271) and left kidney (LK) (*n* = 27 paired LK vs blood DNAmAge donors (C), paired *t*-test: median 69 years vs. median 65 years; *p* = 0.0245) are significantly older than blood leukocytes DNAmAge. RK and LK DNAmAge are similar (A).

Still in Figure 1, AgeAcc of RK and LK present higher score (this mean the AgeAcc is faster) than that of blood leukocytes in the same donor (*n* = 27 RK vs blood AgeAcc donors (E), paired *t*-test: median−5 years vs. median−8 years; *p* = 0.0271, and *n* = 27 LK vs. blood AgeAcc donors (F), paired *t*-test: median−6 years vs. median−7 years; *p* = 0.0245). No difference between RK and LK AgeAcc is observed (D).

**Figure 2:**
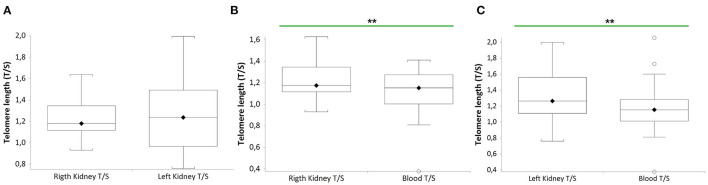
TL of both kidneys (right and left) and blood leukocytes in the same donors (*n* = 27). Box plots show levels of TL in right and left kidneys (RK and LK) **(A)**, in RK and blood **(B)**, and in LK and blood **(C)** of the same donors. In box plots, the boundary of the box closest to the x-axis indicates the 25th percentile, the line within the box marks the mean, and the boundary of the box farthest from the x-axis indicates the 75th percentile. Whiskers (error bars) above and below the box indicate the 95 and 5th percentiles. In **(B)**, the horizontal bar with asterisks indicates the significant comparison between RK TL and paired blood TL of the same donor (*n* = 27) (^**^Paired *t*-test: median 1.18 T/S vs. median 1.15 T/S; *p* = 0.0011). In **(C)**, the horizontal bar with asterisks indicates the significant comparison between LK TL and paired blood TL of the same donor (*n* = 27) (^**^Paired *t*-test: median 1.25 T/S vs. median 1.15 T/S; *p* = 0.0098).

Figure 2 shows that TL of RK and LK are significantly longer than that of blood leukocytes in the same donor (*n* = 27 paired *t*-test: RK TL vs. blood donors (B), median 1.18 T/S vs. median 1.15 T/S; *p* = 0.0011; *n* = 27 LK TL vs. blood donors (C), paired *t*-test: median 1.25 T/S vs. median 1.15 T/S; *p* = 0.0098). No difference between RK and LK TL is observed (A).

**Figure 3:**
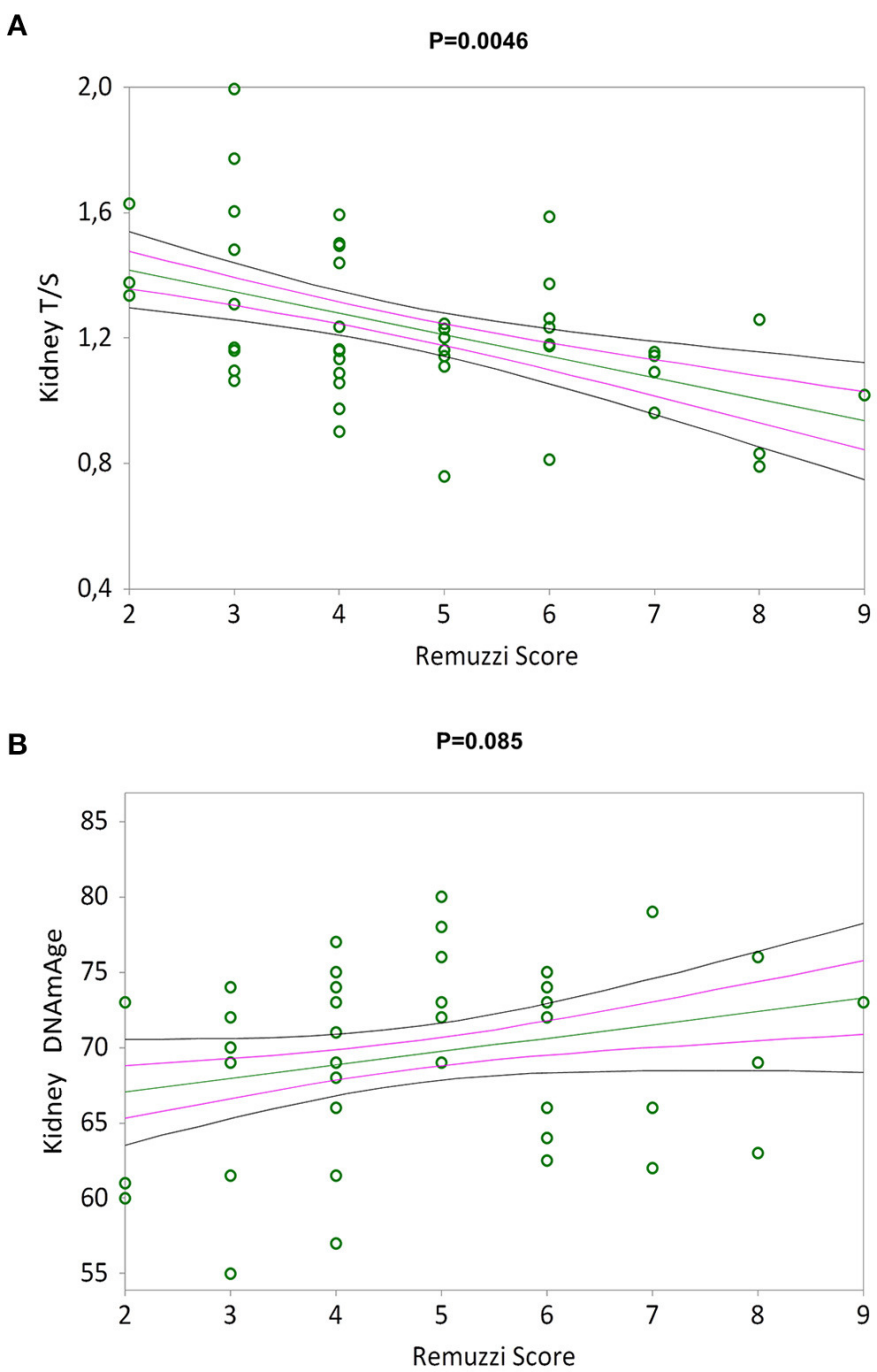
TL and DNAmAge of the kidneys and the Remuzzi-Karpinski score correlations. Non-parametric linear regression plot showing correlations between TL **(A)** and DNAmAge **(B)** of the donors kidneys and the Remuzzi-Karpinski score (Kendall's rank correlation coefficient tau b = −0.310 and Kendall's rank correlation coefficient tau b = 0.192). Mean, Standard Error (SE) and 95% coefficient intervals (CI) are represented as green, pink and black lines, respectively.

Figure 3A shows a negative correlation between the Remuzzi-Karpinski score and kidney TL (Kendall's rank correlation coefficient tau b = −0.310, *p* = 0.0046). Remuzzi-Karpinski score and DNAmAge were instead positive weakly correlated (Figure 3B, Kendall's rank correlation coefficient tau b = 0.192, *p* = 0.085). In Supplementary Table S1, multiple linear regression analysis of the influence of chronological age, gender, kidneys DNAmAge and TL on Remuzzi-Karpinski score of the donors' kidneys, shows that kidneys TL is the main determinant of the Remuzzi-Karpinski score (*p* = 0.0173), but not age, gender and DNAmAge.

In Supplementary Table S2 in supplementary material blood leukocytes DNAmAge, AgeAcc and TL are not related to the leukocytes count in donors.

### Correlation Between Biological Age (DNAmAge, AgeAcc and TL) and Chronological Age, in RK, LK and Blood Leukocytes

**Figure 4:**
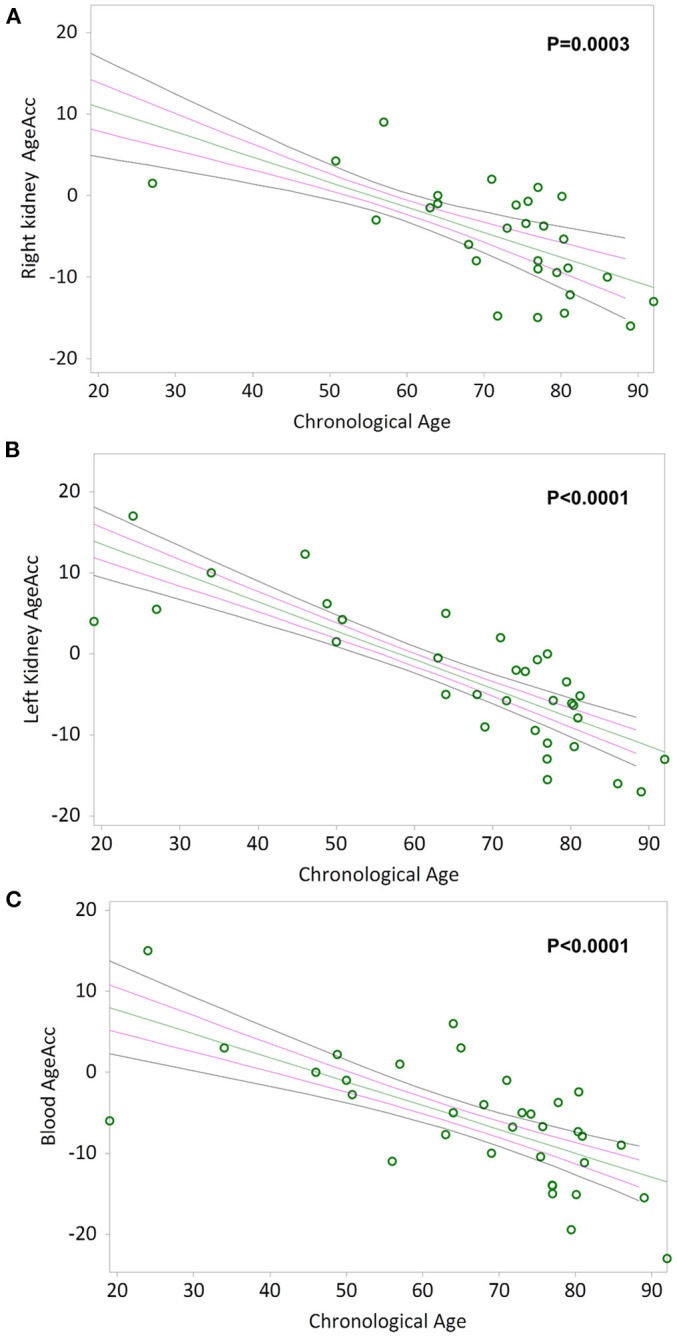
AgeAcc of the right kidney, the left kidney, and blood leukocytes correlation with donors' chronological age. In **(A)** and **(B)**, non-parametric linear regression plots showing the correlation between AgeAcc of RK and LK, and donors chronological age (Kendall's rank correlation coefficient tau b for RK = −0.483 and tau b for LK = −0.638). In **(C)**, non-parametric linear regression plot showing the correlation between AgeAcc of the circulating blood leukocytes (indicated as “blood AgeAcc”) and the donors chronological age (Kendall's rank correlation coefficient tau b = −0.526). Mean, Standard Error (SE) and 95% coefficient intervals (CI) are represented as green, pink and black lines, respectively.

Simple linear regression analyses show that AgeAcc of RK, LK and blood leukocytes are negative correlated with chronological age in Figures 4A–C respectively (Kendall's rank correlation coefficient tau b for RK = −0.483, *p* = 0.0003; LK = −0.638, *p* < 0.0001; blood leukocytes = −0.526, *p* < 0.0001).

DNAmAge of RK, LK and blood leukocytes are positive highly correlated with chronological age (Kendall's rank correlation coefficient tau b for RK = 0.546, LK = 0.663 and blood leukocytes = 0.636, *p* < 0.0001 in Supplementary Figures S1A–C respectively). Instead, TL of RK, LK and blood leukocytes are negative correlated with chronological age in Supplementary Figures S1D–F, respectively (Kendall's rank correlation coefficient tau b for RK = −0.257, *p* = 0.0532; LK = −0.403, *p* = 0.0011; blood leukocytes = −0.277, *p* = 0.0224).

### Correlation Between RK, LK and Blood Leukocytes Biological Age (DNAmAge, AgeAcc and TL)

**Figure 5:**
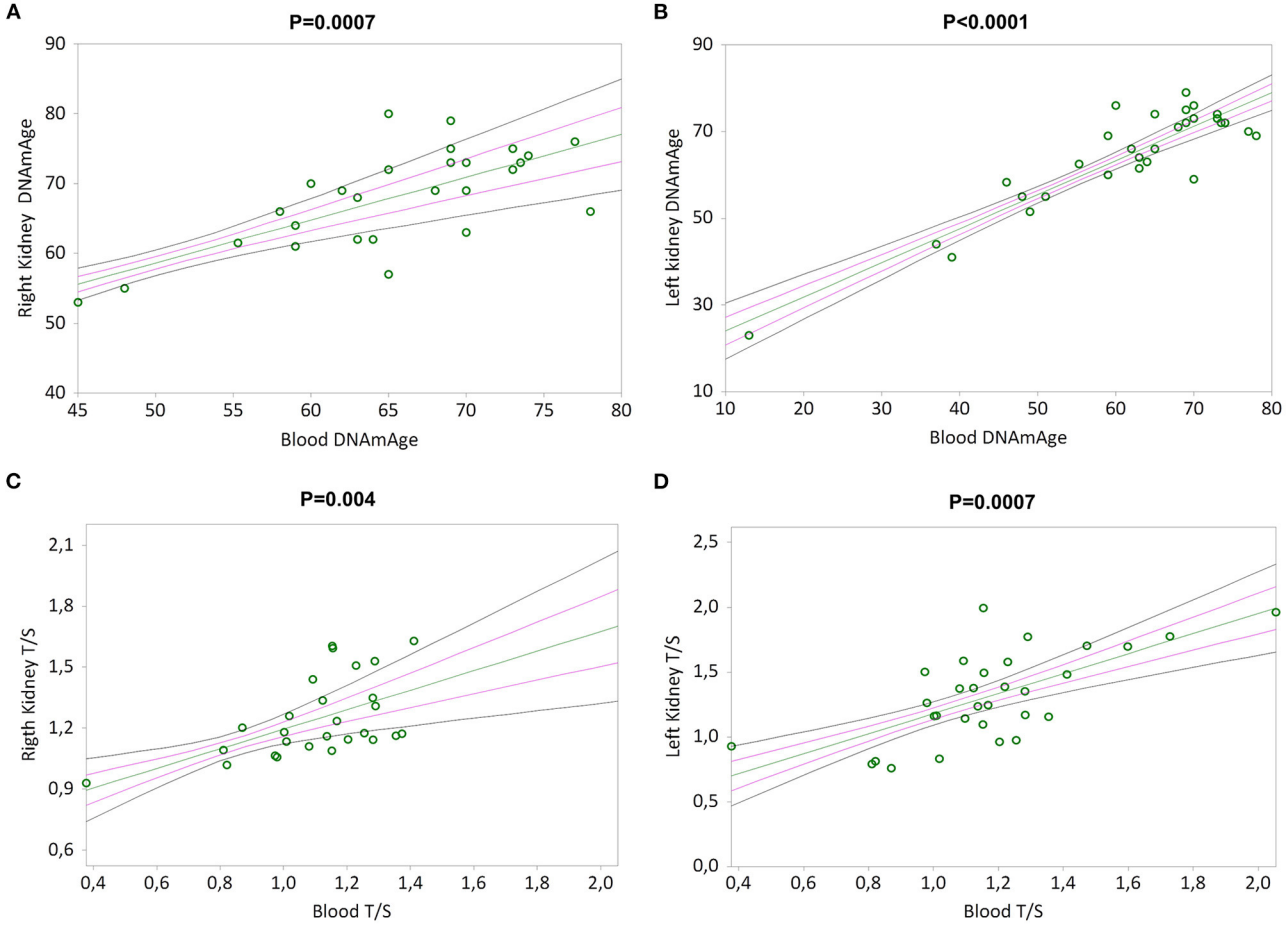
DNAmAge and TL in both kidneys (right and left) correlation with blood leukocytes. In **(A)** and **(B)**, non-parametric linear regression plots showing the correlation between DNAmAge of the right kidney (RK) and the left kidney (LK), and blood DNAmAge (Kendall's rank correlation coefficient tau b for RK = 0.479 and tau b for LK = 0.540). In **(C)** and **(D)**, non-parametric linear regression plots showing the correlation between TL of the right kidney (RK) and the left kidney (LK), and blood TL (Kendall's rank correlation coefficient tau b for RK = 0.396 and tau b for LK = 0.432). Mean, Standard Error (SE) and 95% coefficient intervals (CI) are represented as green, pink and black lines, respectively.

Simple linear regression analyses show that RK and LK DNAmAge correlate with that of blood leukocytes (Kendall's rank correlation coefficient tau b for RK = 0.479, *p* = 0.0007 and LK = 0.540, *p* < 0.0001 in Figures 5A,B), as well as RK and LK TL correlate with that of blood leukocytes (Kendall's rank correlation coefficient tau b for RK = 0.396, *p* = 0.004 and LK = 0.432, *p* = 0.0007 in Figures 5C,D).

Furthermore, the epigenetic non-mitotic DNAmAge and the mitotic age (TL) of RK, LK and blood leukocytes are negatively associated (Kendall's rank correlation coefficient tau b for: RK = –*0*.369, *p* = 0.0057; LK = −0.327, *p* = 0.0083; blood leukocytes = −0.346, *p* = 0.0046 in Supplementary Figures S2A–C respectively).

Age-adjusted TL of RK, LK and blood (calculated as the residual of a regression of TL onto chronological age) were associated with age-adjusted DNAmAge (i.e., AgeAcc). These positive and significant correlations are reported in Supplementary Figures S3A–C, respectively, in Supplementary Materials (Kendall's rank correlation coefficient tau b for: RK = 0.476, *p* = 0.0004; LK = 0.632, *p* < 0.0001; blood leukocytes = 0.537, *p* < 0.0001 in Supplementary Figures S3A–C respectively).

### Determinants of Blood Leukocytes and Kidneys DNAmAge, AgeAcc, and TL

Multiple regression analysis (Supplementary Table S3 in Supplementary Material) of the influence of chronological age (years), gender, leukocytes (10^3^/mL), smoking and chronic diseases including arterial hypertension, diabetes and cancer on blood leukocytes DNAmAge, AgeAcc and TL, shows that the main determinants are chronological age for DNAmAge (*p* < 0.0001) and arterial hypertension for TL (*p* = 0.0014).

Multiple linear regression analysis (Supplementary Table S4 in Supplementary Material) of the influence of creatinine (mg/dL), cancer, Type 2 Diabetes (T2D), arterial hypertension (PAH), smoking, chronological age, suitability of organ for transplantation and gender on kidney DNAmAge, AgeAcc and TL shows that none of the variables considered are related to the DNAmAge, AgeAcc and TL levels. While chronological age is confirmed as determinant of both kidneys DNAmAge (*p* < 0.0001) and kidneys TL (*p* = 0.0011).

## Discussion

In this study, we have determined the biological age of the kidney and of peripheral blood leukocytes of donors, by measuring the non-mitotic epigenetic age (DNAmAge and AgeAcc) and the mitotic age (TL) and correlate it to the Remuzzi-Karpinski score.

The main findings stemming from this work reveal that:

a) The non-mitotic epigenetic age (DNAmAge and AgeAcc) of both kidney tissues (RK and LK) is older than that of blood leukocytes;
b) AgeAcc of both kidney tissues (RK and LK) and blood leukocytes significantly slow down with advancing in chronological age;
c) The mitotic age (TL) of both kidney tissues (RK and LK) is younger than that of blood leukocytes and TL attrition is strongly correlated with the increase in Remuzzi-Karpinski score;
d) DNAmAge, and TL of RK and LK highly correlate with those of blood leukocytes.

DNA methylation is currently the most promising molecular marker for monitoring biological aging and predicting life expectancy (29–31). In humans, DNA methylation changes start early in life, as demonstrated by longitudinal studies of infants' blood (32, 33). Notably, these early epigenetic profiles continue to accumulate changes with the advancement of age, even more so in twins that do not share the same habits and/or environments (34, 35), indicating that aging-associated DNA methylation changes are caused by environmental factors too. In the present study, we demonstrated that epigenetic age (DNAmAge) and AgeAcc of the RK and LK are higher than that of blood leukocytes. This would suggest that the kidney is more susceptible than blood, to epigenetic changes induced by the interaction of advancing age and environmental factors. The aging kidney presents 11.5% of the CpG sites significantly altered (36) in respect to the 0.05–4% of CpG sites reported for other organs (37, 38). A recent report showed that kidney cells make a negligible contribution in terms of the cellular turnover of the human body compared to blood cells and gut epithelial cells (39). Therefore we cannot exclude that the higher methylation in kidney could be attributable to the fact that kidney cells are differentiated and nonproliferative, which allows for the progressive accumulation of epigenetic changes. The hypermethylation correlates with interstitial fibrosis and glomerulosclerosis after kidney transplantation, as well as to reduced renal function (36). One of the causes proposed for biological aging is the accumulation of background noise at the epigenome level, which disrupts the gene expression patterns, leading to a decrease in tissue function and regenerative capacity. A kidney-specific epigenome-wide study of renal biopsies obtained from donors prior to kidney transplantation (range of age: 16–73 years), demonstrated a causal relationship between DNA hypermethylation and age-associated kidney dysfunction (36). Altered genes included those controlling epithelial cell proliferation, susceptibility to apoptosis, stem cell function, and activation of inflammatory cells. The age-related hypermethylation in kidneys is also associated to loss of DNA hydroxymethylation, suggesting a reduced activity of the ten-eleven traslocation (TET) demethylation enzymes, probably due to increased oxidative stress of the aged kidney, drives these age-related changes. Many key factors that influence DNA methylation, including advanced donor age, alloreactive immune responses, ischemia–reperfusion injury, and fibrosis, have the greatest prognostic impact in kidney transplantation, significantly contributing to allograft survival of transplanted patient (40). Therefore, DNA methylation changes represent an interesting research area in kidney transplantation. Mammalian tissues have recently been shown to retain a record of juvenile epigenetic information, encoded by DNA methylation, which can be accessed by acting on methylation to improve tissue function and promote *in vivo* regeneration. In our previous studies, we demonstrated that intensive relaxing training turns back the epigenetic clock (DNA methylation age, DNAmAge), together with improvement in clinical blood parameters (i.e., stress hormones, inflammatory markers, etc.) and with a clinical regaining of endothelial function, in patients after myocardial infarction, and even more in healthy subjects (17). In the whole this suggests that younger epigenetic information can be recovered and indicators of biological clock may represent an accurate tool to measure the effectiveness of interventions. Given the reversible nature of the epigenetic mechanisms, we hypothesize that a demethylation treatment in a kidney under normothermic reperfusion, by also intervening with DNA methylation inhibitors, which may allow restoring normal cellular functions, could be proposed in order to rejuvenate the kidney.

We found that AgeAcc of both kidney and blood leukocytes significantly decreased with advancing chronological age, while we confirmed that DNAmAge are highly correlated with chronological age as previously reported by Horvath (14) and Hannum et al. (15). The reduction in the aging rate of the epigenetic clock in older donors agrees with the hypothesis proposed by Horvath that the ticking rate of the epigenetic clock slows down in later life (14). Furthermore, the rates of epigenetic AgeAcc, has been associated with symptoms of aging, such as frailty and menopause (41, 42), as well as to several aging-associated pathologies including cancer and neurodegenerative diseases (14, 43, 44). AgeAcc can also predict life expectancy independently of common risk factors (19, 45). Nevertheless, the implications of the biological age determination in the field of kidney transplantation have never been explored before. Our results would suggest that AgeAcc might be the epigenetic clock mirroring the real biological state of the kidney. The reduction in the aging rate (AgeAcc) and therefore the slowing down of biological aging in older donors could have a paramount value on the evaluation and use of organs from these donors.

In this study, we also demonstrated that TL of RK and LK were longer than that of blood leukocytes, suggesting that mitotic age of kidney tissue is younger than blood leukocytes. Our results are consistent with the lower cellular turnover in renal cortex/medulla compared to blood leukocytes (39, 46) and to a drop in telomere shortening 9–29 bp/year in kidney cells compared to 41–84 bp/year in blood leukocytes (46). Furthermore, TL of RK and LK highly correlated with chronological age. In this regard, our results are in line with those reported by Melk and colleagues (47) that explored the relationship between age and TL in surgical samples from 24 human kidneys. They found that TL shortens in an age-dependent manner in the kidney with an average of 29 bp/year. We confirmed the inverse correlation between blood leukocytes TL and chronological age that is well-documented in literature. In a systematic review of such association in adults, an almost identical significant negative correlation of about R = 0.3, between mean chronological age and mean LTL, was observed across 124 cross-sectional studies (20).

Furthermore, the rise in DNAmAge and the decline in TL of RK, LK and blood leukocytes were firmly correlated. Studies comparing DNA methylation age and TL in the same sample are few and limited to blood samples (48–52). Our findings are consistent with our previous work on heart donors in which DNAmAge negatively correlated with TL in heart and blood (10), suggesting that DNA methylation and telomeres, even if depending from different mechanisms of the same process (biological aging), they can be associated. Shorter TL is a measure of “mitotic age” also defined as “replicative senescence” (53, 54). Telomeres shorten with every cell division, ticking as a cellular “molecular clock” (55) or a replication “timer.” It was shown that telomeres in highly proliferative somatic tissues are shorter than in cells of non- or low rate proliferative tissues (56). On the other hand, the DNAmAge of differentiated cells does not mirror their proliferative history (12), as evidenced in the Horvarth's epigenetic clock, which produces similar DNAmAge estimation within the same individual for highly proliferative tissues (i.e., blood and colon) and less-proliferative ones (i.e., blood and colon) (14).

We also observed a strong correlation between the increase in Remuzzi-Karpinski score and TL attrition. The Remuzzi score is a histological scoring system obtained at pre-implantation biopsy ranging from 0 (no lesions) to 12 score (marked changes in vessels, glomeruli, tubules and connective tissue) (5). An increased Remuzzi score, being related with poor graft outcomes, is currently used for the decision of discarding donor kidneys or transplanting two kidneys with intermediate score (5, 57). Our finding, showing that kidneys with high Remuzzi score present shorter TL, indicates that such damaged organs present more “mitotic clock miles.” Considering that TL attrition was also associated with decreased graft survival post-transplant (58), this finding acquires further strategic importance in assessing the quality of the organ.

In order to promptly translating our work into the clinical practice, we also assessed the similarity level of biological ages between kidneys and blood leukocytes in the same donor. We observed a robust correlation between DNAmAge and TL of kidneys (RK and LK) and blood leukocytes, suggesting that the latter could be a surrogate tissue of the kidney status for biological aging studies. To the best of our knowledge, such correlation for DNAmAge has not been investigated before now. Determining the biological age of blood and estimating its correlation with that of the kidneys is of paramount importance to identify and set up a simple and reliable tool for screening potential donors, in particular for accepting the kidneys for transplantation from elderly donors. In a real clinical scenario, at the donor hospital site, blood samples may be easily and quickly acquired and sent to the laboratory for biological age analysis. Further studies are hence needed to optimize the use of blood as a surrogate indicator of kidney's biological age in clinical practice.

Furthermore, the slowing down of biological aging (AgeAcc) in older donors could be a key parameter in the evaluation and use of organs from these donors. For example, we can hypothesize to define a cutoff of AgeAcc, beyond which the donor becomes at risk of graft dysfunction rather than considering the value of the chronological age.

Our study for the first time evaluated the non-mitotic epigenetic age and the mitotic age from kidneys of donors by comparing it with that of their blood leukocytes in healthy donors. This pioneering aspect certainly represents one of the main strengths of our study. Analyzing DNAmAge and TL by using an almost totally automated workflow, which allows us to perform the analyses in a standardized way reducing errors, is another strong point of this study. Furthermore, no significant difference was found in the distribution of RK and LK DNAmAge, and in TL as well. Our study for the first time evaluates the non-mitotic epigenetic age (DNAmAge) and mitotic age (TL) of donor kidneys, and levels were similar for both kidneys. This suggests that, in the future, biological age analysis can be performed either in the RK or LK. Lastly, our results showed that kidneys TL and DNAmAge correlate with those of blood leukocytes, suggesting that blood could be a more easily and quickly acquired surrogate tissue of the kidney for biological aging studies for translating into the clinical practice.

A limitation of our study could be the small number of subjects enrolled and number of samples collected, which was due to the limited available donors on which biopsies can be performed due to clinical reasons. However, samples analyzed are adequate from the statistical point of view. Another limitation could be that renal biopsy, from which we extracted DNA for biological age analysis, contains different cell types. However, up to now, renal biopsy remains the gold standard by which essential diagnostic and prognostic information is obtained in kidney transplantation. Biopsy methodologies have been developed to assess the acceptability of an organ before transplantation and to assess and predict renal allograft performance after implantation (2, 5). In addition, the donor organ biopsy sample provides a valuable baseline against which the results of subsequent biopsies of the renal allograft can be compared (2, 5). Furthermore, the few studies regarding the analyses of telomere and methylation profiles in the kidney were performed on kidney biopsies, as well as our study. However, recent advances in single cell genomics are transforming researchers' ability to characterize single cells (59). Incorporation of these new tools into traditional histopathologic evaluation of renal tissue is needed to improve diagnostic precision and predictive value of the renal biopsy in kidney disease. Therefore, further studies are needed in this field.

Another limitation could be that DNAmAge measure used in this study was composed of only 5 CpG sites while other DNAmAge measures are composed of up to several hundred CpG sites. However, amongst the most robust predictors, we selected the model proposed by Zbieć-Piekarska et al. (16). This method shows that DNAmAge highly correlates (r = 0.94) to chronological age with a mean deviations from calendar age (4.5 years) analogous to those from Horvath (14) and Hannum et al. (15) (r = 0.96 and r = 0.91) with 3.6 and 4.9 years mean considered the reference methods. Zbieć-Piekarska et al. (16) developed the algorithm in a larger sample (*n* = 420) and then they validated it in a smaller one (*n* = 300), covering the entire adult life span. Furthermore, to increase the practicability of these tests, and used the locus-specific technology pyrosequencing which having the potential for multiplexing, makes the technical analysis achievable in few hours and reduce the cost of technical analysis, methods with only few loci were taken into account. Furthermore, we have automated to increase the efficiency and rapidity, maintaining high prediction accuracy (9, 10). Lastly, analyzing DNAmAge by using this process in an almost totally automated workflow, we can perform the analyses in a standardized way reducing errors, and this is a strong point of our study.

In conclusion, main findings stemming from our study are that the epigenetic age of kidney was older than that of the blood leukocytes, but the mitotic age measured by TL of kidney is younger than that of blood leukocytes suggesting that, in view of the low cellular turnover of the kidney cells, they are more susceptible than blood to epigenetic changes, which allows for the progressive accumulation of background noise at the epigenome level. Therefore, given the reversible nature of the epigenetic mechanisms of DNA, in the current era of shortage of organs, methylation changes might represent an interesting research area, which could offer a potential translation into kidney transplantation. Our finding, showing that kidneys with high Remuzzi score present shorter TL, indicates that such marker can be evaluated in further studies as markers of donor organ quality. Our study although deserves future focused investigations on post-transplant graft performance and durability in relation to the biological age, could contribute to open a novel basic and clinical research platform in the field of all solid organs' transplantation.

## Data Availability Statement

The raw data supporting the conclusions of this article will be made available by the authors, without undue reservation.

## Ethics Statement

The studies involving human participants were reviewed and approved by the Ethical Committee for Clinical Trials of the Province of Padova. The patients/participants provided their written informed consent to participate in this study.

## Author Contributions

SP and MC: conceived and designed the study and provided administrative, technical, or material support (i.e., reporting or organizing data, constructing databases). LF, FN, MB, PR, and EN: provided the samples. MC: performed the samples' analysis. SP and MC: analyzed the data. SP, MC, and FN: wrote the paper. All authors contributed to the article and approved the submitted version.

## Funding

This study was supported by the funding Grants the L.I.F.E.L.A.B., Consorzio Per La Ricerca Sanitaria-CORIS (Veneto Region).

## Conflict of Interest

The authors declare that the research was conducted in the absence of any commercial or financial relationships that could be construed as a potential conflict of interest.

## Publisher's Note

All claims expressed in this article are solely those of the authors and do not necessarily represent those of their affiliated organizations, or those of the publisher, the editors and the reviewers. Any product that may be evaluated in this article, or claim that may be made by its manufacturer, is not guaranteed or endorsed by the publisher.

## Abbreviations

AgeAcc: Age acceleration
DNAmAge: DNA methylation age
LK: Left Kidney
LTL: Leucocytes telomere length
RK: Right Kidney
TL: Telomere length.
