# Supplementary material for: DNA Methylation - and Telomere - Based Biological Age Estimation as Markers of Biological Aging in Donors Kidneys
Source: Front Med (Lausanne). 2022 Mar 23;9:832411. doi: 10.3389/fmed.2022.832411 (PMC8984253; doi:10.3389/fmed.2022.832411)
Supplement: Supplementary file 1 [file Data_Sheet_1.docx]

**Title: DNA Methylation - and Telomere - based biological age estimation as markers of biological aging in donors kidneys.**

**Manuela Campisi^1**^, Flavia Neri^2^, Paolo Rigotti^2^, Lucrezia Furian^2^, Sofia Pavanello^1*^**

^1^ Occupational Medicine, Department of Cardiac, Thoracic, and Vascular Sciences and Public Health, University Hospital of Padova, Via Giustiniani, 2, 35128, Padova, Italy.

^2^ Kidney and Pancreas Transplantation Unit, Department of Surgery, Oncology and Gastroenterology, University Hospital of Padova, Via Giustiniani, 2, 35128, Padova, Italy

*Corresponding author

Prof. Sofia Pavanello

Department of Cardiac, Thoracic, Vascular Sciences and Public Health, University of Padova

Via Giustiniani 2, 35128 Padova, Italy;

e-mail: [sofia.pavanello@unipd.it](mailto:sofia.pavanello@unipd.it)

*Corresponding authors ORCID ID <https://orcid.org/0000-0002-5229-9900>

**ORCID ID <https://orcid.org/0000-0002-7372-4136>

**Supplementary Material**

**1 Materials and Method**

**1.1 Donor’s kidney biopsy procedure**

Over a time span of 22 months (from March 2019 to January 2021) renal true-cut biopsies samples were obtained from 36 deceased kidney donors for whom the procurement was performed by the surgical team of Kidney and Pancreas Transplantation Unit - Department of Surgical, Oncological and Gastroenterological Sciences, University Hospital of Padua. The donors included in the study were those for whom a renal biopsy was deemed clinically indicated either for the assessment of chronic damage (1) or for the presence of acute kidney injury. After the incision, abdominal organs were examined and the vessels prepared for the perfusion. After the subdiaphragmatic aorta crossclamp, 7 to 10 Liters of cold (4°C) Celsior solution was flushed through a cannula into the abdominal aorta to perfuse and preserve all the organs. After the procurement of the liver and of the pancreas, if suitable, the kidneys were procured en-bloc and divided at the bench on ice. After removal of the perirenal fat, the kidney parenchyma was assessed and a sample was taken at the upper pole with a 16G needle semi-automatic biopsy gun.

The tru-cut biopsies were placed in all protected tissue reagent-RNA Later (Qiagen, Milano, Italy) for DNA/RNA stabilization. All collected samples were then, transferred to our laboratory of Genomic and Environmental Mutagenesis (Department of Cardiac, Thoracic, and Vascular Sciences and Public Health, University-Hospital of Padua) for genetic and epigenetic analyses and stored at −20 °C, until analyses were performed.

**1.2 DNA extraction from blood and tissue samples**

DNA extraction was performed on all samples of whole blood and renal biopsies using an automated QIAcube System (Qiagen, Milano, Italy) according to the DNAeasy Blood and Tissue kit (Qiagen, Milano, Italy) procedure for high-throughput purification, following the manufacturer’s instructions and customized protocols as previously described (2). Briefly, 400 µL of whole blood were processed for DNA extraction, while an average amount of 4.8 mg of tissue sample, obtained from the renal biopsies, was disrupted using TissueLyser II (Qiagen, Milano, Italy). This system, through high-speed shaking with beads, beats and grinds samples, that release biomolecules and simultaneously homogenize samples to facilitate subsequent DNA purification procedure. After extraction, all DNA samples were quantified and checked for quality and integrity using QIAexpert Quantification System (Qiagen, Milano, Italy). We obtained genetic material suitable for subsequent analytical procedures from a qualitative point of view (mean 260/280 = 1.91).

**1.3 DNAmAge analysis**

DNAmAge was determined by analyzing the methylation levels of five selected markers (ELOVL2 C1orf132, KLF14, TRIM59 and FHL2) in genomic DNA using bisulfite conversion and Pyrosequencing methodology as previously described (2–4), with some modifications relatively to the set-up of an almost completely automated method by using the PyroMark Q48 Autoprep Autoprep (Qiagen, Milano, Italy). In brief, 1 μg of DNA extracted from both blood and renal tissue samples, was submitted to bisulfite conversion using Epitect Fast DNA Bisulfite (Qiagen, Milano, Italy) to convert unmethylated cytosines into uracil, following manufacturer’s instructions. An aliquot of template DNA was used for PCR amplification of selected markers using PCR primers included in the AgePlexMono kit (Biovectis, Warszawa, Poland). PCR reactions were performed in 25 μL, comprising 0.2 μM of each primers, 20 ng of [template DNA](https://www.sciencedirect.com/topics/medicine-and-dentistry/dna-template), and PyroMark PCR Master Mix holding HotStarTaq [DNA Polymerase](https://www.sciencedirect.com/topics/medicine-and-dentistry/dna-polymerase), 1x PyroMark PCR Buffer and dNTPs. The amplification plan involved a preliminary [denaturation](https://www.sciencedirect.com/topics/biochemistry-genetics-and-molecular-biology/denaturation) step at 95°C for 10 min, followed by 40–45 cycles of denaturation (94°C for 30 s), annealing (54–56°C for 60 s) and extension (72°C for 90 s), and a final extension of 72°C for 10 min. Each PCR amplification contained negative PCR controls. Finally, 10 µl of PCR products were used for each pyrosequencing primer contained in AgePlex Mono kit (Biovectis, Warszawa, Poland) and loaded in a 48 well-plate (Pyromark Q48 Discs, Qiagen, Milano, Italy). Details on PCR and sequencing primer sequences and the sequences analyzed are given in Table S1. Pyrosequencing was performed on the Pyromark Q48 Autoprep instrument (Qiagen, Milano, Italy)using Pyromark Q48 Advanced Reagents (Qiagen, Milano, Italy) according to manufacturer’s instructions. The resulting Pyrograms generated by the instrument were automatically analyzed using Pyromark Q48 Autoprep Software (Qiagen, Milano, Italy). The methylation percentages of methylated cytosines at the 5 CpG sites were inserted in an online calculator system accessible at [www.agecalculator.ies.krakow.pl](http://www.agecalculator.ies.krakow.pl), for estimation of biological age from DNA methylation analysis. Twenty percent of the samples were analyzed in two different days to verify the reproducibility of our results and the coefficient of variation (CV) in replicate pyrosequencing runs was 1.7 %.

**1.4 TL analysis**

TL was measured after DNA extraction from both whole blood and renal needle biopsies samples, by using quantitative Real-Time PCR as previously reported (5,6). This assay measures relative TL in genomic DNA by estimating the ratio of telomere repeat copy number (T) to single nuclear copy gene (S) in experimental samples relative to the T/S ratio of a reference pooled sample. The single-copy gene employed in this study was human (beta) globin (hbg) (5,6). A “seven- point” reference curve was built from a serially diluted DNA pool, varying from 40 to 0.625 ng/ µl in each plate, in order to calculate the relative quantities of T and S in ng of samples to be examined. The DNA pool was realized for both blood samples and renal biopsies samples, by taking an aliquot of genomic DNA from samples at random designated. Briefly, Qiagility (QIAGEN, Milano, Italy) was used for transferring 10 μl of reaction mix and 5 μl of DNA (5 ng/μl) in a 96-well plate. The PCR reactions were performed on a StepOnePlus Real-Time PCR System (Applied Biosystems, Monza, Italy).

A primer pair for a beta‐globin single copy gene (HBG1: GCTTCTGACACAACTGTGTTCACTAGC;HBG2: CACCAACTTCATCCACGTTCACC) as well as a telomere primer pair (Telg: ACACTAAGGTTTGGGTTTGGGTTTGGGTTTGGGTTAGTGT; Telc: TGTTAGGTATCCCTATCCCTATCCCTATCCCTATCCCTAACA) were used in the reaction mix. The thermal cycling profile for both amplicons began with incubation at 95°C for 210 min to activate the AmpliTaq DNA polymerase. For telomere PCR, activation was followed by 2 cycles of 15 sec at 95°C, 15 sec at 49°C, and 35 cycles of 15 sec at 95°C, which was then followed by 10 sec at 62°C, and 15 sec at 74°C. For hbg, activation was followed by 35 cycles of 15 sec at 95°C and 1 min at 58°C. The efficiency of the PCR was accepted if between 90–100% (−3.6 ≥ slope ≥ −3.3). TL was analyzed in triplicate for all samples and standards. The average of the three T measurements was divided by the average of the three S measurements to calculate the average T:S ratio, i.e. the relative telomere length. A measure was considered acceptable if the standard deviation (SD) among triplicate measures was <25%. TL was also replicated in different days on 60% of the total samples, thus minimizing the effect on the measurements related to the well position and plate. The average of coefficient of variation for the T:S ratio of blood and tissue samples analyzed over three consecutive days was 9%, which was similar to the reproducibility originally reported for this method (7). Furthermore, the resulting interclass correlation coefficient (ICC), an indicator of measurement reliability, was 0.75 indicating good reliability.

**References**

1. Pierobon ES, Sefora PE, Sandrini S, Silvio S, De Fazio N, Nicola DF, et al. Optimizing utilization of kidneys from deceased donors over 60 years: five-year outcomes after implementation of a combined clinical and histological allocation algorithm. *Transpl Int.* (2013) 26:833–841. doi:10.1111/tri.12135

2. Pavanello S, Campisi M, Fabozzo A, Cibin G, Tarzia V, Toscano G, et al. The biological age of the heart is consistently younger than chronological age. *Sci Rep.* (2020) 10:10752. doi:10.1038/s41598-020-67622-1

3. Pavanello S, Campisi M, Tona F, Lin CD, Iliceto S. Exploring Epigenetic Age in Response to Intensive Relaxing Training: A Pilot Study to Slow Down Biological Age. *Int J Environ Res Public Health.* (2019) 16:E3074. doi:10.3390/ijerph16173074

4. Campisi M, Liviero F, Maestrelli P, Guarnieri G, Pavanello S. DNA Methylation-Based Age Prediction and Telomere Length Reveal an Accelerated Aging in Induced Sputum Cells Compared to Blood Leukocytes: A Pilot Study in COPD Patients. *Front Med (Lausanne).* (2021) 8:690312. doi:10.3389/fmed.2021.690312

5. Pavanello S, Stendardo M, Mastrangelo G, Bonci M, Bottazzi B, Campisi M, et al. Inflammatory Long Pentraxin 3 is Associated with Leukocyte Telomere Length in Night-Shift Workers. *Front Immunol.* (2017) 8: doi:10.3389/fimmu.2017.00516

6. Pavanello S, Campisi M, Mastrangelo G, Hoxha M, Bollati V. The effects of everyday-life exposure to polycyclic aromatic hydrocarbons on biological age indicators. *Environmental Health.* (2020) 19:128. doi:10.1186/s12940-020-00669-9

7. Cawthon RM. Telomere length measurement by a novel monochrome multiplex quantitative PCR method. *Nucleic Acids Res.* (2009) 37:e21. doi:10.1093/nar/gkn1027

**Supplementary Tables**

**Table 1S** Multiple regression analyses of the influence of chronological age (years) and gender (F=1; M=0), kidneys DNAmAge and TL on Remuzzi-Karpinski score of the donors’ kidneys.

|  | **Variables** | **b** | **r** | **t** | **p-Value** |
| --- | --- | --- | --- | --- | --- |
| ***Remuzzi-Karpinski score*** | Chronological Age | b1 = 0.032762 | r = 0.116642 | t = 0.752005 | p = 0.4563 |
|  | Gender (M=0; F=1) | b2 = 0.028283 | r = 0.008656 | t = 0.055427 | p = 0.9561 |
|  | Kidneys DNAmAge | b3 = -0.021683 | r = -0.059993 | t = -0.384835 | p = 0.7023 |
|  | Kidneys TL | b4 = -2.8354 | r = -0.361286 | t = -2.480936 | **p = 0.0173** |

**Table 2S**. Influence of leukocytes count (10^3^/ml) on DNAmAge, AgeAcc and TL of blood leukocytes in donors.

| Variables | DNAmAge | | | AgeAcc | | | TL | | |
| --- | --- | --- | --- | --- | --- | --- | --- | --- | --- |
|  | *b* | *r* | *p-Value* | *b* | *r* | *p-Value* | *b* | *r* | *p-Value* |
| Leukocytes (10^3^/ml) | 0.117238 | 0.053721 | 0.7628 | 0.082898 | 0.067162 | 0.7059 | 0.003509 | 0.075516 | 0.6712 |

**Table 3S.** Multiple regression analyses of the influence of chronological age, gender, leukocytes (10^3^/mL), smoking, Type 2 Diabetes (T2D), arterial hypertension (PAH) and cancer on blood leukocytes DNAmAge, AgeAcc and TL.

|  | **Variables** | **b** | **r** | **t** | **p-Value** |
| --- | --- | --- | --- | --- | --- |
| ***DNAmAge*** | Chronological Age | b1 = 0.690802 | r = 0.863201 | t = 8.718222 | **p < 0.0001** |
|  | Gender (M=0; F=1) | b2 = -1.85747 | r = -0.159311 | t = -0.822837 | P = 0.4181 |
|  | Leukocytes (10^3^/mL) | b3 = 0.172452 | r = 0.162276 | t = 0.838562 | p = 0.4094 |
|  | Smoking (No=0; Yes=1) | b4 = 0.553023 | r = 0.038366 | t = 0.195775 | p = 0.8463 |
|  | Arterial Hypertension (PAH) (No=0; Yes=1) | b5 = 2.165466 | r = 0.130434 | t = 0.670818 | p = 0.5083 |
|  | Type 2 Diabetes (T2D) (No=0;Yes=1) | b6 = -2.598856 | r = -0.130879 | t = -0.673143 | p = 0.5068 |
|  | Cancer (No=0; Yes=1) | b7 = -3.740311 | r = -0.167128 | t = -0.864347 | p = 0.3953 |
| ***AgeAcc**** | **Variables** | **b** | **r** | **t** | **p-Value** |
|  | Gender (F=1; M=0) | b1 = -2.218656 | r = -0.151432 | t = -0.796044 | p = 0.433 |
|  | Leukocytes (10^3^/mL) | b2 = 0.068536 | r = 0.052272 | t = 0.271986 | p = 0.7877 |
|  | Smoking (No=0; Yes=1) | b3 = 2.607526 | r = 0.144786 | t = 0.760342 | p = 0.4536 |
|  | Arterial Hypertension (PAH) (No=0; Yes=1) | b4 = -3.856072 | r = -0.20721 | t = -1.100581 | p = 0.2808 |
|  | Type 2 Diabetes (T2D) (No=0; Yes=1) | b5 = -1.649724 | r = -0.066534 | t = -0.346489 | p = 0.7317 |
|  | Cancer (No=0; Yes=1) | b6 = -9.06615 | r = -0.32516 | t = -1.786672 | p = 0.0852 |
| ***TL*** | **Variables** | **b** | **r** | **t** | **p-Value** |
|  | Chronological Age | b1 = -0.001282 | r = -0.171988 | t = -0.890233 | p= 0.3815 |
|  | Gender (F=1; M=0) | b2 = -0.051232 | r = -0.102698 | t = -0.526443 | p = 0.603 |
|  | Leukocytes (103/mL) | b3 = -0.010103 | r = -0.219678 | t = -1.148189 | p = 0.2613 |
|  | Smoking (No=0; Yes=1) | b4 = -0.206138 | r = -0.319859 | t = -1.7214 | p= 0.0971 |
|  | Arterial Hypertension (PAH) (No=0; Yes=1) | b5 = -0.437663 | r = -0.573977 | t = -3.574086 | **p = 0.0014** |
|  | Type 2 Diabetes (T2D) (No=0; Yes=1) | b6 = 0.269451 | r = 0.296494 | t = 1.58301 | p = 0.1255 |
|  | Cancer (No=0; Yes=1) | b7 = -0.34752 | r = -0.355965 | t = -1.942295 | p = 0.063 |

*The variable Age is not considered for AgeAcc because of its own definition.

Bold character is displayed only for significant values.

**Table 4S.** Multiple regression analyses of the influence of creatinine (mg/dL), cancer, Type 2 Diabetes (T2D), arterial hypertension (PAH), smoking, chronological age, suitability of organ for transplantation and gender on kidney DNAmAge, AgeAcc and TL.

|  | **Variables** | **b** | **r** | **t** | **p-Value** |  |
| --- | --- | --- | --- | --- | --- | --- |
| ***DNAmAge*** | Creatinine (mg/dL) | b1 = 0.148074 | r = 0.045645 | t = 0.23299 | p = 0.8176 |  |
|  | Cancer (No=0; Yes=1) | b2 = 0.249173 | r = 0.012039 | t = 0.061394 | P = 0.9515 |  |
|  | Type 2 Diabetes (T2D) (No=0; Yes=1) | b3 = -0.888626 | r = -0.05897 | t = -0.301214 | p = 0.7656 |  |
|  | Arterial Hypertension (PAH) (No=0; Yes=1) | b4 = 1.630548 | r = 0.12321 | t = 0.633072 | p = 0.5322 |  |
|  | Smoking (No=0; Yes=1) | b5 = -3.546085 | r = -0.285556 | t = -1.519317 | p = 0.1408 |  |
|  | Chronological Age | b6 = 0.594591 | r = 0.879518 | t = 9.424244 | **p < 0.0001** |  |
|  | Suitability of organ for transplantation (No=0; Yes=1) | b7 = 2.37302 | r = 0.210111 | t = 1.095822 | p = 0.2832 |  |
|  | Gender (F=1; M=0) | b8 = -0.376914 | r = -0.040305 | t = -0.205683 | p = 0.8386 |  |
| ***AgeAcc**** | **Variables** | **b** | **r** | **t** | **p-Value** |  |
|  | Creatinine (mg/dL) | b1 = -0.387864 | r = -0.074837 | t = -0.389958 | p = 0.6996 |  |
|  | Cancer (No=0; Yes=1) | b2 = -4.460919 | r = -0.134983 | t = -0.70787 | p = 0.4851 |  |
|  | Type 2 Diabetes (T2D) (No=0; Yes=1) | b3 = -0.486345 | r = -0.020097 | t = -0.104449 | p = 0.9176 |  |
|  | Arterial Hypertension (PAH) (No=0; Yes=1) | b4 = -6.133554 | r = -0.312284 | t = -1.708102 | p = 0.0991 |  |
|  | Smoking (No=0; Yes=1) | b5 = 0.184396 | r = 0.009943 | t = 0.051669 | p = 0.9592 |  |
|  | Suitability of organ for transplantation (No=0; Yes=1) | b6 = 2.337312 | r = 0.130453 | t = 0.683697 | p = 0.5 |  |
|  | Gender (F=1; M=0) | b7 = -1.266528 | r = -0.084196 | t = -0.439057 | p = 0.6641 |  |
| ***TL*** | **Variables** | **b** | **r** | **t** | **p-Value** |  |
|  | Creatinine (mg/dL) | b1 = -0.032836 | r = -0.224926 | t = -1.177065 | p = 0.2498 |  |
|  | Cancer (No=0; Yes=1) | b2 = 0.098645 | r = 0.107957 | t = 0.55371 | p= 0.5845 |  |
|  | Type 2 Diabetes (T2D) (No=0; Yes=1) | b3 = -0.076503 | r = -0.11509 | t = -0.590771 | p = 0.5598 |  |
|  | Arterial Hypertension (PAH) (No=0; Yes=1) | b4 = -0.125204 | r = -0.212241 | t = -1.107453 | p = 0.2782 |  |
|  | Smoking (No=0; Yes=1) | b5 = -0.202247 | r = -0.361039 | t = -1.974096 | p = 0.0591 |  |
|  | Chronological Age | b6 = -0.010135 | r = -0.583085 | t = -3.659675 | **p = 0.0011** |  |
|  | Suitability of organ for transplantation (No=0; Yes=1) | b7 = -0.034527 | r = -0.071056 | t = -0.363235 | p = 0.7194 |  |
|  | Gender (F=1; M=0) | b8 = -0.030432 | r = -0.073992 | t = -0.378326 | p = 0.7083 |  |

*The variable Age is not considered for AgeAcc because of its own definition.

Bold character is displayed only for significant values.

**Supplementary Figures**


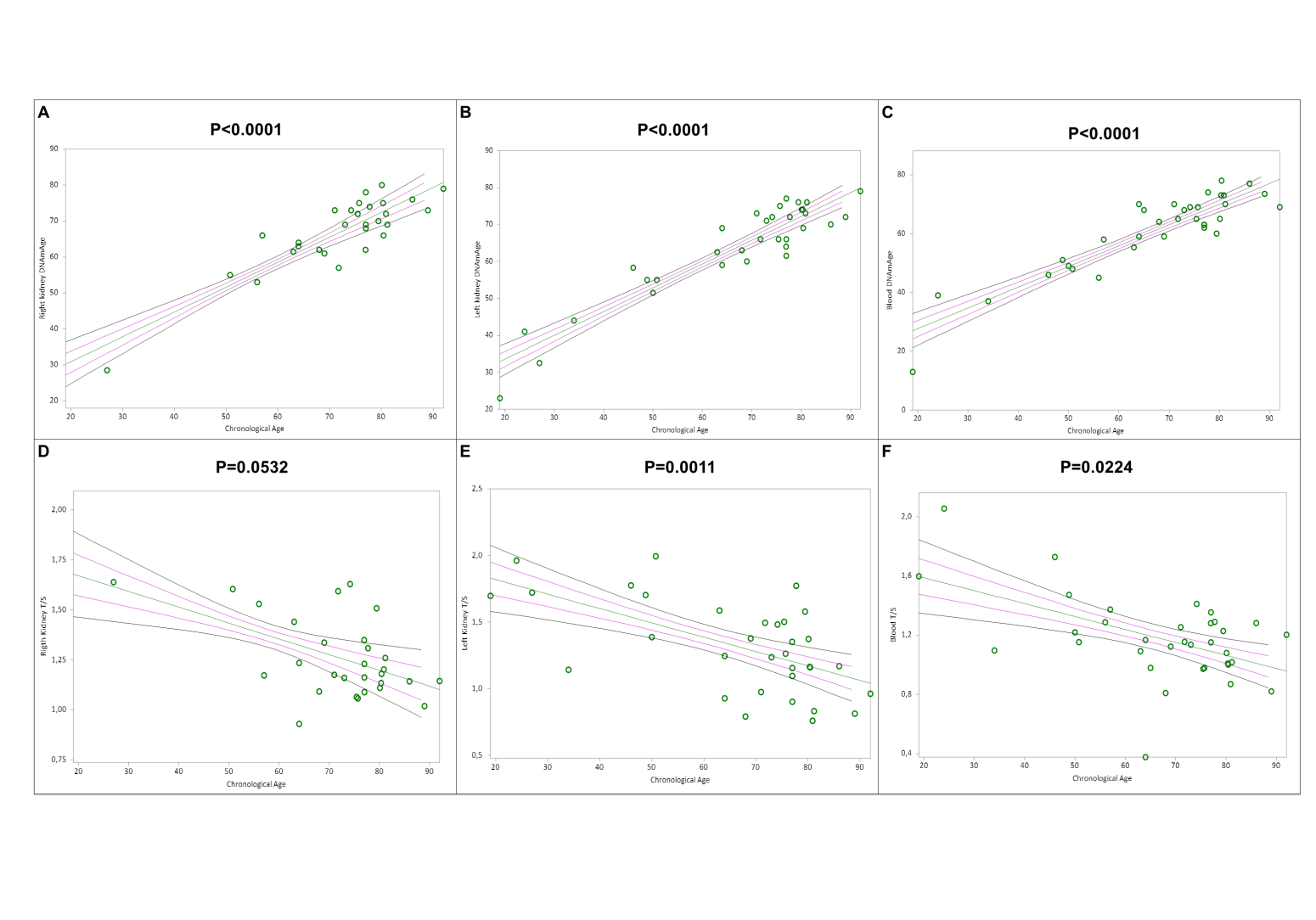


**Figure 1S. Correlation curves between DNAmAge or TL of the right kidney, the left kidney, and blood leucocytes with donors’ chronological age.**

In (**A**) and (**B**), non-parametric linear regression plots showing correlation between DNAmAge of the right kidney (RK) and the left kidney (LK) and donors chronological age (Kendall’s rank correlation coefficient tau b for RK = 0.546, for LK = 0.663); whereas in (**C**), non-parametric linear regression plot showing the correlation between DNAmAge of the circulating blood leucocytes (indicated as “blood DNAmAge”) and the donors chronological age (Kendall’s rank correlation coefficient tau b = 0.636 ).

In (**D**) and (**E**), non-parametric linear regression plots showing correlation between telomere length (T/S) of the right kidney (RK) and the left kidney (LK) and donors chronological age (Kendall’s rank correlation coefficient tau b for RK = - 0.257, for LK = - 0.403 ); whereas in (**F**), non-parametric linear regression plot showing the correlation between telomere length (T/S) of the circulating blood leucocytes (indicated as “blood T/S”) and donors chronological age (Kendall’s rank correlation coefficient tau b = - 0.277 ).

Mean, Standard Error (SE) and 95% coefficient intervals (CI) are represented as green, pink and black lines, respectively.


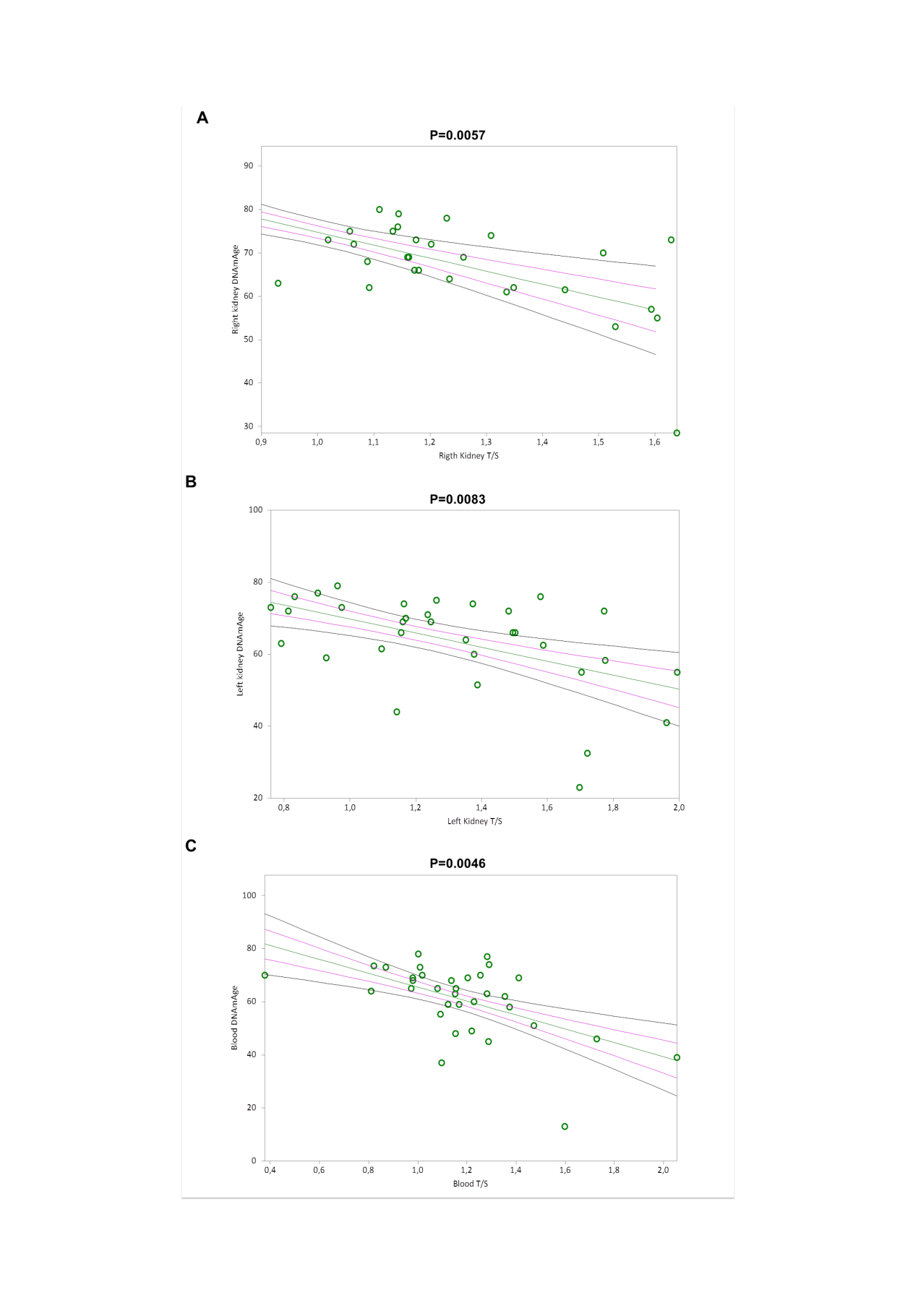


**Figure 2S. Correlation curves between DNAmAge and TL of the right kidney, left kidney, and blood leucocytes.**

In (**A**) and (**B**), non-parametric linear regression plots showing correlation between DNAmAge and telomere length (T/S) of the right kidney (RK) and the left kidney (LK) (Kendall’s rank correlation coefficient tau b for RK = -0.369, for LK = -0.327). In (**C**), non-parametric linear regression plot showing the correlation between DNAmAge and telomere length (T/S) of the circulating blood leucocytes (indicated as “blood DNAmAge and blood T/S”) (Kendall’s rank correlation coefficient tau b = - 0.346).

Mean, Standard Error (SE) and 95% coefficient intervals (CI) are represented as green, pink and black lines, respectively.

**
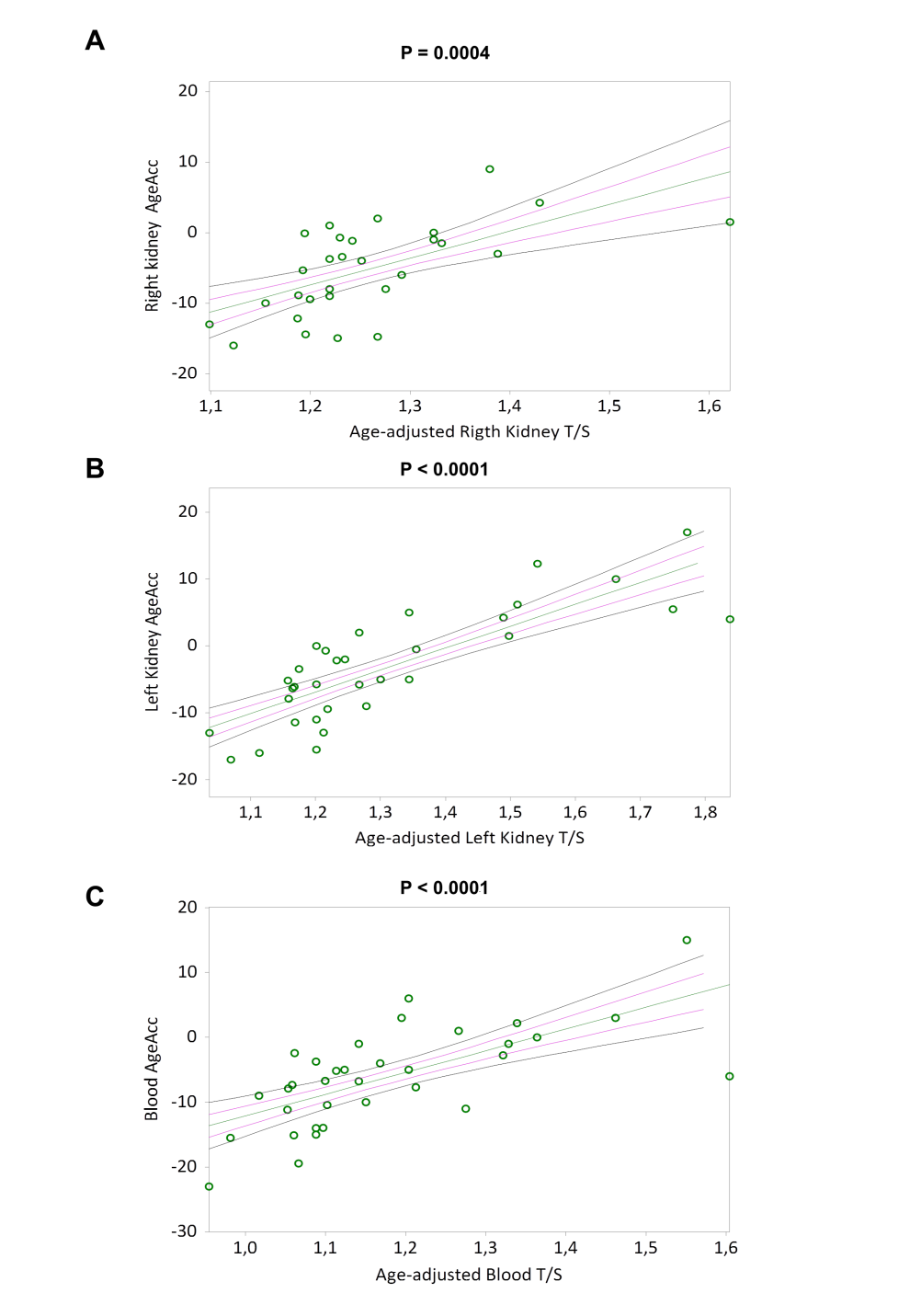
**

**Figure 3S.** **Correlation curves between AgeAcc and age-adjusted TL in right and left kidneys and in blood leukocytes.**

In (**A**) and (**B**), non-parametric linear regression plots showing correlation between AgeAcc (years) and age-adjusted TL (T/S) in right (R-) and left kidneys (LK) (Kendall’s rank correlation coefficient tau b for RK = 0.476, for LK = 0.632). In (**C**), non-parametric linear regression plot showing the correlation between AgeAcc (years) and age-adjusted TL (T/S) of the circulating blood leukocytes (indicated as “blood AgeAcc and Age-adjusted Blood T/S”) (Kendall’s rank correlation coefficient tau b = 0.537).

Mean, Standard Error (SE) and 95% coefficient intervals (CI) are represented as green, pink and black lines, respectively.
